# Anti-COVID-19 Vaccine Narratives Among African and North American Neo-Pentecostals (Part 1): Evidence, Causes and Lessons

**DOI:** 10.1007/s10943-025-02556-4

**Published:** 2026-01-19

**Authors:** Daniel Orogun

**Affiliations:** 1 Department of Religion Studies, University of Pretoria, Pretoria, South Africa; 2 Centre for Studies in Religion and Society, University of Victoria, British Columbia, Canada; 3 UC Davis Medical Centre, University of California, Davis, Sacramento, USA

**Keywords:** Anti**-**vaccine narratives, Spiritual leaders, Theology of medicine, Health behavior, Africa and North America, Neo-Pentecostals

## Abstract

The gap in the worldview of spiritual leaders regarding public health and safety became apparent during the COVID-19 pandemic. It engendered narratives and health behaviors that led to the deaths of many. This interdisciplinary study examines the relationship between religious beliefs, practices, and health behavior. In two parts, this empirical research, through a literature review, secondary data analysis, and available narratives in academic and media spaces, deconstructs the activities of some African and North American Neo-Pentecostals during the pandemic. Part 1, presented here, provides evidence of the narratives and discusses the causes of misinformation and hesitancy. Part 2 addressed the public health implications, the ideal theological response, leadership gaps, and lessons learned. Three fundamental points are evident in both parts. The first is a poor “theology of medicine” in health crises. The second is the poor leadership approach to crisis management. The third is the long-term tension and a lack of synergy between health professionals, health policymakers, and spiritual leaders. The outcome of Part 1 revealed that the causes of anti-vaccination narratives are rooted in theological, social, and economic factors. Part 1 concludes with a summary of the lessons and an overview of what to expect in Part 2 of the exercise.

## Introduction

In the author’s journey as an interdisciplinary researcher focusing on the interaction of spirituality, healthcare and health systems, one of the captivating scriptures arguing in favor of the role of spiritual leadership in public health has been 3 John 2: “Beloved, I pray that in every way you may prosper and enjoy good health, as your soul also prospers—BSB” (YouVersion, 2025). In two words and phrases—*beloved, every way, good health,* and *prosper*—this scriptural verse describes the ideal biblical worldview of Christian leaders and adherents vis-à-vis personal, community, and public health.

The text above suggests that the creator’s intention is for humans to prosper by utilizing every means possible to achieve good health, including adhering to provincial or national healthcare protocols and encouraging vaccination to prevent community health calamities. This worldview does not seem to be the initial approach of many Neo-Pentecostals in Africa and America during the recent COVID-19 pandemic. Some of the spread of the virus associated with “religious events” was confirmed as early as February 2020, and the risk of large-scale religious events being pandemic triggers was also highlighted (Sisti et al., 2023, 1–20; Ebrahim, 2020, 395). Besides death, the impact of such events includes grief, community trauma in churches, and nations.

This article focuses on spiritual leadership during the pandemic for three critical purposes. First, there are ongoing discussions about the possibility of another outbreak in a few years. Some researchers contend that the likelihood of another pandemic in the next four years is 10–15%, and the world is unprepared (Dyos, 2025). The Southeast Asia report by Bajeli-Datt shows that the COVID pandemic may return in 5 years (Bajeli-Datt, 2025).

Second, the world has not fully recovered from the events of 2020–2021. An epidemiologist from the Sidney School of Public Health asserts that the virus that caused the pandemic, SARS-CoV-2, is still circulating and there are still high chances of hospitalization of patients with COVID (Sheel, 2025). Thirdly, the purpose of reviewing the stories of death, anti-vaccine claims, public health implications, and leadership failure is to respond to an important call from Pastor Terris King of Liberty Grace Church of God in Baltimore. In the Frontiers of Health and Medicine journal, Sokolow interviewed Terry King. The clergy challenge interdisciplinary researchers to tell stories of health and spirituality to improve awareness and quality responses to vaccinations, as follows.

“I think that the nexus of religion and health care has not been examined, utilized, and exhausted in the African American community.” …I don’t think there has been enough attention to the importance of storytelling, who those storytellers are, and the effectiveness of utilizing those storytellers to build a bridge between health care institutions and the community.” (Sokolow, 2020,1).

This article takes the above statements by Terris King seriously as a call to tell the stories of loss and gains of religious leaders and groups’ response to health crises during the pandemic. Telling the stories and showing some leadership’s theological pitfalls are instrumental to preparing for the eventual emergence of another pandemic. These would be done in two parts. This current article serves as Part 1. It will present academic literature, secondary research data, and media narratives as evidence, supported by case studies from Africa and North America. An extensive discussion about the causes of misinformation from theological, social, and economic perspectives will follow this. A summary will be presented in the conclusion, including lessons from the narrative and what to expect in Part 2.

## Brief Literature Reviews and Narratives

The intent to maintain brevity in this section is predicated on the need to weave more narratives into theological and public health conversations in the following sections.

**Fig. 1:**
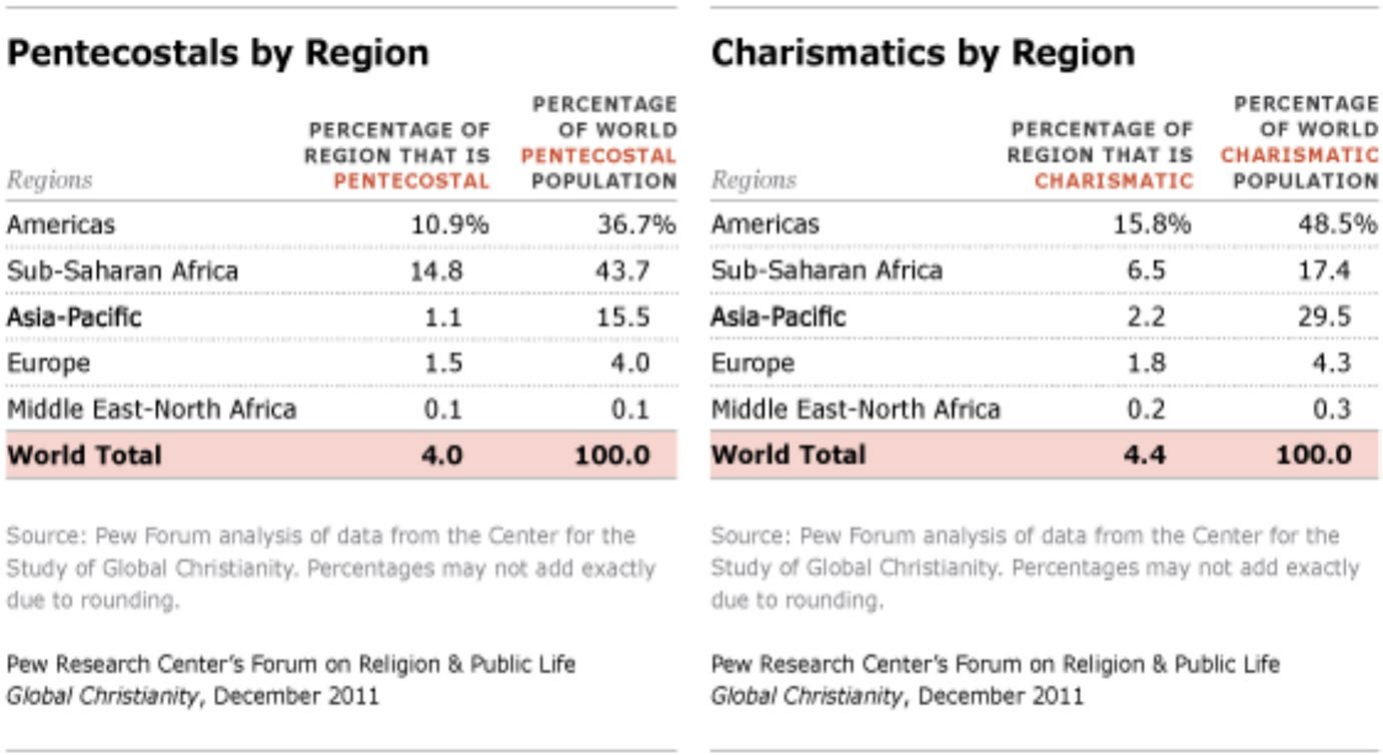
Pentecostals by Region. Source: Liu, 2011, Pew Research Centre

Before proceeding to the narratives, it is essential to discuss the selection of Neo-Pentecostals in Africa and North America as the focus of this study. The largest Pentecostal population in the world is concentrated in America and sub-Saharan Africa, as demonstrated in Fig. 1. Such a high population suggests the possibility of higher risks and impacts of non-compliance with health protocols and anti-vaccine narratives in communities and nations.

**Fig. 2:**
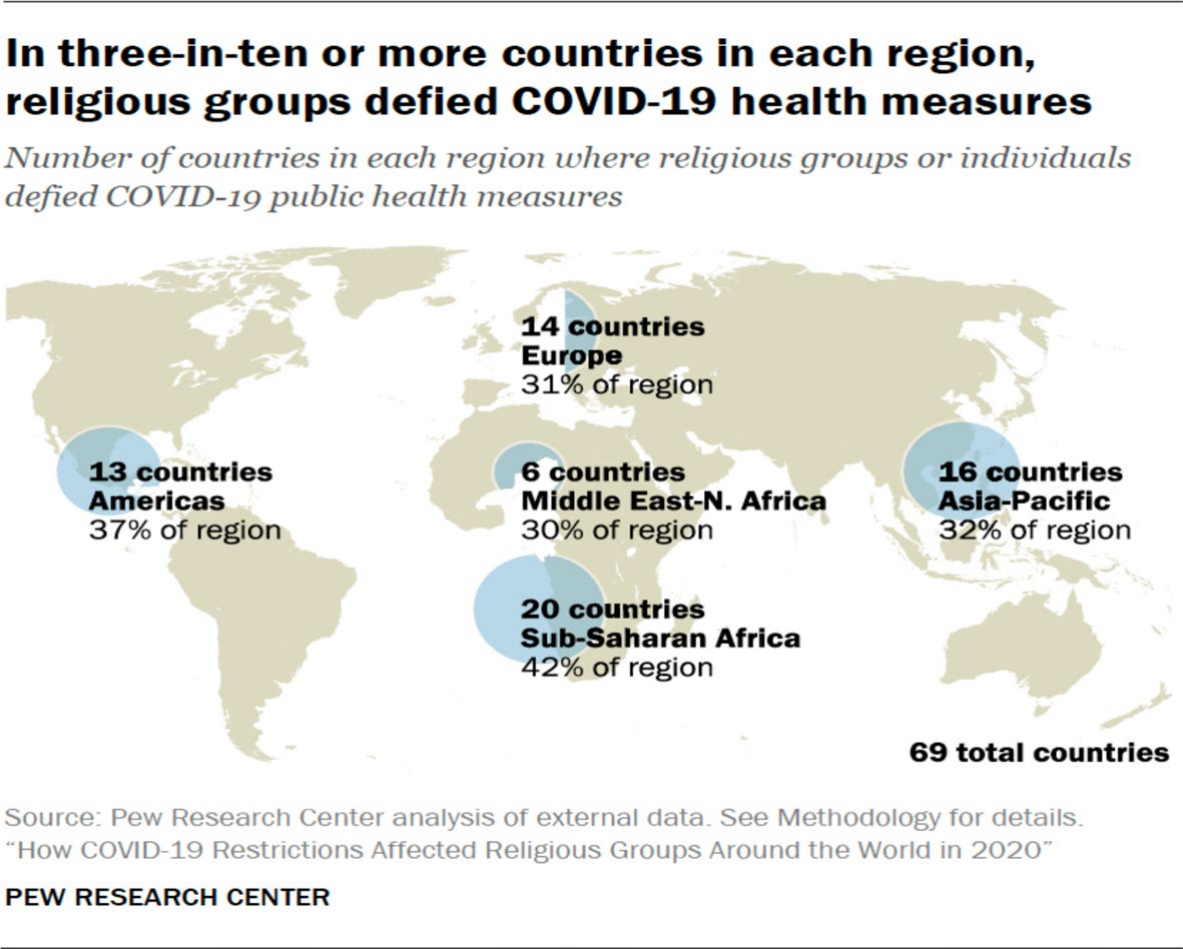
Regions where religion defied health measures. Source: Majumdar, 2022a, Pew Research Centre

According to Pew Research data, Africa and the USA were most resistant to governments’ health authority protocols (Majumdar, 2022b). Figure 2 shows countries with the highest percentages of religious groups and individuals who defied public health measures during the COVID-19 pandemic. The argument here is that the Neo-Pentecostal Christian groups have the highest population where vaccination and resistance to health protocols have occurred.

With Africa and America having 42% and 37% of religious defiance to healthcare measures during the pandemic, as seen in the data above, telling the stories and investigating the loopholes is necessary to reduce the percentage in any eventual recurrence. Beginning with Africa, the following stories will be presented below, with additional narratives outlined in the next section (Narratives II).

### Africa

In the Free State province in South Africa, a single religious event attended by three COVID-19-positive church leaders led to the infection of more than 80 people and the further tracing of 1600 people who may have been exposed to the virus (Sisti et al., 2023, 12–13*;* Jaja et al., 2020). These religious leaders defied health protocols by continuing religious gatherings and pushing back on vaccination. Such actions contributed to vaccination hesitancy and death.

Earlier research shows that hesitancy about vaccinations did not start with COVID-19. A study in Zimbabwe shows the prevalence of hesitancy and rejection of measles and rubella vaccines based on religious teachings that emphasize prayer as an alternative to vaccines, and on the lack of privacy and personal rights to decision on vaccination in a religiously controlled community. Such an alternative reinforces the hesitancy generated by poor knowledge of vaccine safety and effectiveness among members of the Apostolic Church (Machekanyanga et al., 2017, 84).

Likewise, Pastor Frankline Ndifor of Kingship International Ministries placed no significance on the vaccine in Cameroon. The preacher despised the health protocols and vaccinations, but focused more on miraculous healing. Pastor Frankline was convinced he could heal anyone with a COVID-19 infection by laying hands on them (The Christian News, 2020; Kindzeka, 2020). Rather than allow congregants to pursue vaccination and follow health protocols during the pandemic, the pastor chose to promote miraculous healing of COVID-19 through the laying of hands. Unfortunately, Frankline died of the same infection.

As the African nation with the highest number of Pentecostal adherents and pastors, Nigeria remains an area that captures the people’s religious beliefs and social imagination. Neo-Pentecostal and charismatic leaders are considered first responders in the line of protection for many Nigerians. Some leverage their authority to serve as existential micromanagers, freely and in some cases rashly making statements on matters of life and death. They speak and prophesy about politics and the economy, and act as spiritual and prophetic physicians who prescribe alternatives to medicine.

A perfect example is the failed COVID-19 prophecy of late Temitope Balogun Joshua, a popular Nigerian Televangelist (Obadare, 2022). He claimed there would be heavy rainfall in China during the pandemic; the rain would wash away the virus, and the pandemic would be over. Indeed, there was heavy rainfall, but the pandemic remained. Although the prophecy failed, the misinformation may have determined the health behavior of many adherents who rejected vaccination.

The following section will explore more literature and narratives regarding defiance to vaccination and hesitancy. In the meantime, the North American narratives are briefly provided below.

### North America

***CANADA*** – In Parkland County, Alberta, Pastor James Coates and the Grace Life Church were charged with violating Alberta’s Public Health Act in late 2020 and early 2021. During the pandemic, the church continued to hold services, filling the hall with people far beyond the expected capacity, violating COVID-19 rules. Coates spent 35 days in jail in 2021 after refusing to agree to the condition of his release, which required him to obey the government’s Public Health Act (Black, 2021; Ellingson, 2023).

In the accused's testimony at the court, Coates claimed to have initially followed public health restrictions but became convinced that COVID-19 was overblown and that public health measures went too far after researching what the preacher termed “real facts.” Coates stated: “**I don’t believe that COVID-19 poses a serious health risk to our people**” (Black, 2021, 1). Coates further describes COVID-19 as a “**so-called pandemic**” (Black, 2021, 1).

The pastor of the First United Pentecostal Church in Bishop’s Falls, Newfoundland, Canada, continued to gather congregants during the COVID-19 pandemic. Members were also unwilling to receive the vaccination. Most members claimed that vaccines may kill more people than COVID-19 itself. The daughter of a deceased faithful eighty-three-year-old woman who never missed a church service lamented that services should never have been held, and the pastor should have acknowledged the increasing spread of the virus in the province. The daughter claims the congregation never observed COVID-19 protocols, and the church leadership provided no strict follow-up on compliance. The complainant claims, **“It could have been avoided…As a leader of a church, he should have stepped up. And having these services did not help”** (Barry, 2021,1).

According to Poitras’ report, one Mr. Gee, whose family members were victims, claims the church is the source of the COVID-19 pandemic outbreak in his family. Likewise, Pickett in Poitras (2021) claims to be aware of at least three churches where pastors have preached against vaccines and other public health measures, such as using masks and social distancing. Gee in Poitras further claims, **“Most of the people I find that are unvaccinated have some tie to one of the churches”** (Poitras, 2021, 1).

Gee further notes that over 20 people who are sick or in the hospital or dead were connected to non-compliance with COVID-19 protocols by a church in Limestone Siding (Poitras, 2021). Correspondingly, Premier Blaine Higgs of the province of New Brunswick agrees that churches played a significant role in the increased cases of COVID-19 in the province. Provincial leadership had to take drastic measures, including filing a civil lawsuit against any pastor whose anti-vaccination preaching was found to have contributed to the outbreak in Carlingford (Poitras, 2021). Another Canadian megachurch pastor, Leon Fontaine, who refused to adhere to the COVID-19 lockdown and continued holding in-person worship, reportedly died at age 59 due to COVID-19 infection (Gryboski, 2022).

***USA –*** In Arkansas, a pastor and more than 30 attendees in a religious ceremony were infected, leading to three related deaths and the infection of over 26 others in the community (Sisti et al., 2023, 12; James et al., 2020, 632–635). Danny Reeves, a pastor in Corsicana, Texas, refused to be vaccinated. Reeves later fought for his life at the Baylor Medical Center ICU in Dallas. With regret after recovery, Reeves joined the “get the vaccine campaign” (Elliott, 2021).

Furthermore, the popularity of unofficial COVID-19 treatment, vaccine hesitancy, and resistance was due to spiritual leaders’ belief that God’s protection through spiritual rituals remains the only practical resource (Sisti et al., 2023, 13). The US “Religion and the Vaccine Survey,” conducted in March 2021, highlights that approximately 50% of Protestants and Mormons were the least vaccine-receptive religious groups.

**Fig. 3:**
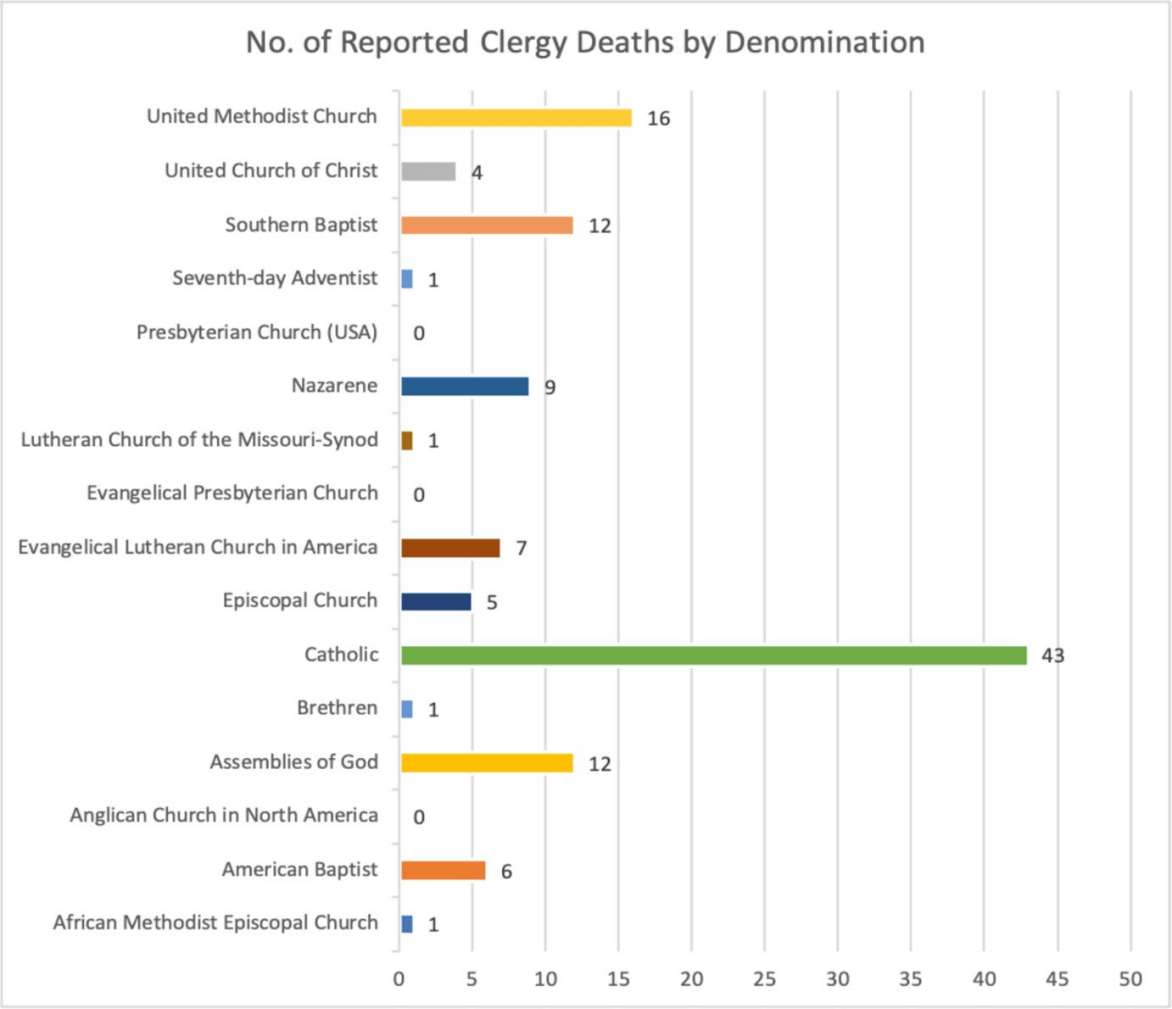
Faithx Project’s Preliminary Summary from 16 different denominations, Source: Howard, 2021

**Fig. 4:**
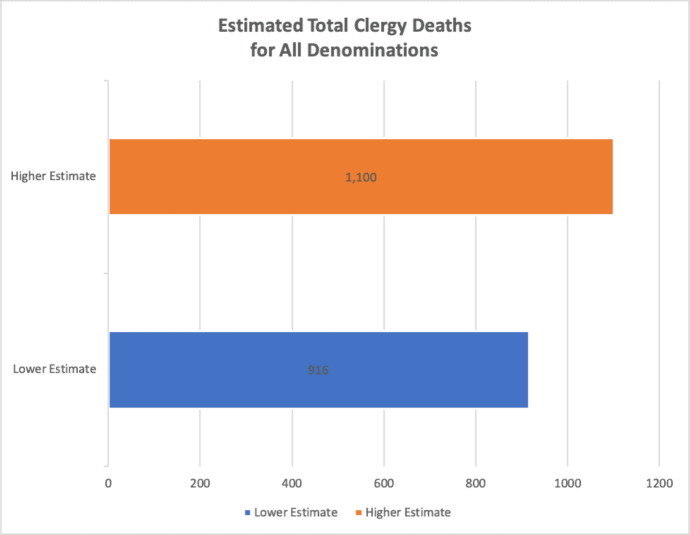
Faithx Project’s Estimated Covid-19-related Clergy Death in America, Source: Howard, 2021

One key gap in this data is the absence of Pentecostals. Howard provides the bridge to the gap in a data submission that shows the Assemblies of God as a Pentecostal church with about the third-highest number of clergy deaths via COVID-19 complications. Howard submits preliminary data that reveals the Christian clergy death in the USA at 118 people, of which Assemblies of God had a death rate of 10.17% which was 12 of 118 deaths. As presented in Fig. 3, the preliminary report shows a lower statistic, which does not speak to a robust argument on the deaths of Pentecostal clergy. To make room for a balanced argument, Howard provided an estimated report, as shown in Fig. 4, since COVID-19 was ongoing at the time of the research.

In support of the estimated report, Dixon asserts, “there will almost certainly be more clergy who die from Covid-19 as time progresses” (Dixon, 2021, 249). Dixon leveraged the WHO report, which forecasted a weekly increase of 149,642 COVID-19 cases and 854 deaths in the USA as of August 16, 2021 (WHO, 2021). Based on this weekly estimation, Howard, in a post-COVID-19 context, then claims, **“If there exists roughly 458,000 Christian clergy members in the entire United States, then as many as 1,100 (high estimate) or 916 (low estimate) clergy members have died as a direct result of COVID-19”** (Howard, 2021). Assuming this high statistical estimation is accurate, juxtaposing the high estimate of 1,100 deaths with the preliminary statistics of 10.17% deaths among the Assemblies of God's clergy would mean a total of 111.87 Assemblies of God clergy died in America during the pandemic. See Figs. 3 and 4.

Beyond Howard’s data, another piece of evidence that Pentecostals were deeply involved in the anti-vaccination narrative is the news from the Church of God in Christ (COGIC), headquartered in South Memphis. As one of the nation’s largest African American Pentecostal denominations, the church suffered a significant leadership loss during the COVID-19 pandemic. Reports show that up to 30 key leaders, including pastors, bishops, and COGIC members nationwide, lost their lives (Mattingly, 2020; Peagler, 2020).

So far, the literature and statistics in this section are evidence of anti-COVID-19 narratives and the consequent resistance to vaccination and healthcare protocol during the pandemic. This raises the question of the influence behind such health behavior. This article aims to address such a question by identifying some causes or sources of misinformation and claims surrounding COVID-19 and vaccinations.

## Causes of Misinformation and Claims: Narratives II

**i. Historical and Social Causes of Anti-Vaccine Narratives and Hesitancy:**

Hesitancy and anti-vaccine did precede the COVID-19 pandemic. There are historical accounts that support the cultural, religious, racial, and political dimensions of vaccine hesitancy and rejection in both Africa and America. The intersection point of Africa and America’s histories of vaccine hesitancy and rejection is trust. Both African and American societies rarely trust Big Pharma and associated groups handling vaccine production and recommendations. Perspectives from both regions will be discussed below.

### America

- ***Christian Nationalism***

Anti-vaccine sentiments found the root of their skepticism in racial, cultural, and religious beliefs. Additionally, vaccination suffers setbacks due to the historical abuses perpetrated by medical professionals and Big Pharma (O’Donnell, 2020; Washington, 2008). A few scholars submit that **“Anti-vaccination skepticism of white evangelical Protestants, political conservatives, and anti-science Americans is an ideological view that seeks to return an exclusivist religious traditionalism into the public sphere and grant epistemic primacy to community authorities—what we and others call Christian nationalism”** (Whitehead & Perry, 2020, 1–2; cf. Gorski, 2017). Further, based on findings, Whitehead and Perry state the following.

“Christian nationalism is the second strongest predictor of general anti-vaxx attitudes...Christian nationalism strongly predicts Americans’ skepticism toward the trustworthiness of doctors and pharmaceutical companies, an elevated assessment of the risks involved, misinformation about the link between vaccines and autism, and belief in parents’ ultimate authority to withhold vaccines from their children” (Whitehead & Perry, 2020, 2).

Christian nationalists believe that science represents a competing epistemic authority; therefore, it encroaches on public life with moral consequences. Some researchers think the tensions between Christian nationalism and science, including medical and pharmaceutical sciences, are “primarily issues about status politics” (Baker et al., 2020, 587). In other words, Christian nationalism, as a significant cultural mechanism connecting politics and religion, strongly opposes science. Rather than relying on scientists and government intervention to address collective problems, such as vaccination-related diseases, Christian nationalists uphold cultures that sustain the moral tenets of the USA, thereby ensuring social and economic stability (Whitehead & Perry, 2020).

- ***Race***

Another social predictor of anti-vaccine attitude is race. The history of racial discrimination and supremacy in the USA supports this assertion. Whitehead and Perry argue that **“One of the most consistent sociodemographic predictors of vaccine uptake and acceptance in the United States is race”** (Whitehead & Perry, 2020, 2). In a further argument, the duo submits that **“Research consistently demonstrates that, compared with whites, black populations are much less likely to receive vaccines”** (Whitehead & Perry, 2020, 2). They do not trust government, healthcare providers, and pharmaceutical companies (Constantine & Jerman, 2007; Galbraith et al., 2016; Webb et al., 2018).

Furthermore, a few authors assert that **“The effects of the fraught history of institutionalized medicine, vaccines, and the African American community are still with us today”** (Washington, 2008, 1). With white supremacy, white Americans are likely confident in the government health programs, including vaccines, whereas Blacks and Hispanics suspect the motive vis-à-vis racial eradication or population reduction (Whitehead & Perry, 2020, 8; Freimuth et al., 2017; Washington, 2008). On account of existential threats and the determination for self-preservation, some Americans develop an anti-vaccine attitude over the years.

- ***Trust***

Regardless of race, North Americans have a long-standing history of distrusting the government and Big Pharma. Some Americans believe the political class and Big Pharma have motives to control the social and cultural way of life of the people. Consequently, they oppose a few healthcare initiatives to preserve social and cultural freedom. Some Americans want to express their faith in public spaces. They consider vaccination and healthcare policies threats to such freedom. Some parents believe that vaccines cause autism, and drug companies are not sincere in disclosing the risks of vaccines to the public (Whitehead & Perry, 2020).

Another matter is the question of safety and authority over children’s well-being. Parents want to decide on vaccination for their children. They generally believe that too many vaccines are being administered to children’s bodies. Parents are concerned that vaccines do not protect children from dangerous diseases and dislike that their decisions are limited in child vaccination matters (Delehanty et al., 2018). In the quest to stop the dominance of government and Big Pharma, they push back on vaccinations. Many of the Christian nationalists hold this position. Therefore, it is consistent to say that Christian nationalism, trust, and race are historically significant predictors of vaccine behavior in America’s public life.

### Africa

The issue of trust is prevalent not only in North America but also in historical examples where Africans have resisted healthcare initiatives from the West. Such trust issues relate to population and cultural preservation, including their religions. This article considers resistance to family planning, polio, and other immunizations as classic examples below.

- ***Family Planning***

In the report following the World Population Conference in Bucharest in 1974, most African governments expressed little support for population control. They held that development is the best contraceptive. This position is primarily influenced by the intent to preserve religious and moral childbirth traditions, counter possible population reduction motives of the West, and reduce poverty driven by underdevelopment (National Research Council [US] Working Group, 1993, 7).

Besides the government officials' efforts, the position of the Catholic, Anglican, and Pentecostal churches condemned modern contraception early enough. For example, the encyclical *Casti Connubii* of Pope Pius XI (1930), encyclical *Humanae Vitae* of Pope Paul VI (1968), apostolic exhortation *Familiaris Consortio* of Pope John Paul II (1981), and speech of Pope Benedict XVI (2008) did not support modern contraception practices but allowed certain traditional contraception for natural rhythms of women’s fertility, like periodic abstinence, cervical mucus, temperature method, etc. (Garenne, 2018).

Like the Catholics, Pentecostal theology and practice do not favor family planning or any related science for population reduction in this period. Likewise, some Muslim schools of thought oppose sterilization and abortion. Highly politicized Islamic groups pushed back on population policy aimed at reducing the Muslim population. These positions vary between countries, schools of thought, and periods (National Research Council [US] Working Group, 1993, 17).

Religion is an essential component of daily life for many people worldwide, with 88.3% of the world’s population associating with a faith, of which 31.6% are Christian and 25.2% Muslim. Concurrently, religion plays a significant role in African life and culture (National Research Council [US] Working Group, 1993, 7). This is why faith leaders are often the most trusted and influential members of African society. The population relies on spiritual leadership to provide direction in controversial cases like abortion and contraception.

For example, a study in Ethiopia shows that over 80% of religious leaders in Islam and Christianity do not subscribe to family planning (Zewoldi & Eca, 2025). These leaders may pass their theology of birth control to their congregants and communities alike. Another study among the Yorubas in Nigeria found that among 81 Christian leaders and 40 Muslim leaders who participated in the research, 12% of Christians and 78% of Muslims had preached against family planning (Orubuloye et al., 1993, 10).

In recent times, the situation in Sierra Leone shows that while Catholics generally opposed interference with procreation on account of religious doctrines, some Pentecostals and Muslims do likewise but permit such interference under certain circumstances to promote family well-being, spacing births, and related health reasons (Yillah et al., 2024, 1). In summary, the history of distrust about population control, from a religious and social perspective, created some foundation for anti-vaccination narratives and hesitancy in Africa.

- ***Polio***

Polio vaccine hesitancy from history is primarily a trust issue (Carrieri et al., 2023). In early 2023, a vaccine hesitancy survey in Ghana, Kenya, Nigeria, South Africa, Tanzania, Uganda, covering 5203 adults, concerning polio (nOPV2), human papillomavirus vaccines (Gardasil4, Cervarix) and COVID-19 (Comirnaty), shows vaccine hesitancy can be transferred from one disease and vaccine contexts to another (Unfried & Priebe, 2024, 1). In other words, the historical vaccine hesitancy surrounding polio in the late twentieth century is transferable to the vaccine hesitancy of the twenty-first century. This was evident in the anti-COVID-19 vaccine narratives.

A report shows a high prevalence of polio in Nigeria, which accounted for 45% of reported cases worldwide and 80% in Africa as of 2003 (Renne, 2006). Earlier in 1996, the Pfizer Trovan trial on 200 children later led to the death of a few and the rejection of the polio vaccine by the Northern Muslim community (Jegede, 2007, 419–420). The situation increased the suspicions about Western health interventions, which were already circulating in northern Nigeria.

In justifying the continued push back on the polio vaccine, notwithstanding the alarming 30% increase in polio incidence, the Kano State Government in January 2004 argued it is a lesser of two evils, to sacrifice a few children to polio than allow millions of girls likely to be rendered infertile by vaccinations (Jegede, 2007, 420; Wise, 2001). Later, Nigerian Muslims worked with Indonesia, a trusted Muslim country, to test the polio vaccine and later resumed their polio vaccination program. Subsequently, about 150 Muslim clerics and traditional chiefs from Chad, Cameroon, Niger, Togo, Benin, and Burkina Faso gathered in Kano on September 22, 2004, to chart a course for the immunization campaign. This indicates a distrust and pushback on vaccination support from Western countries (Jegede, 2007).

Further, Datti Ahmed, the then president of Nigeria’s Supreme Council for Sharia Law, raised allegations that the polio immunization vaccines were contaminated with anti-fertility drugs, HIV/AIDS enabling elements, and Simian virus that can cause cancer. Further insinuation was that the US government and Big Pharma plan to limit Nigeria’s population by spreading AIDS and infertility (Chen, 2004, 206). Although it was not established, part of the narrative was that the polio vaccine contained HIV, cancerous, and anti-fertility agents (Jegede, 2007, 420). These narratives were strong enough to scare families and communities, thereby strengthening their hesitancy toward vaccinations.

- ***Other Immunizations***

Vaccine hesitancy is historical and has been a transference that has shifted into the media space. Reports show that one-fifth of South African social media users are vaccine-hesitant (Wiysonge, 2019). In the 1990s (late twentieth century), Cameroonians opposed child immunization because vaccinations were being used to sterilize girls. In the same period, an investigation reveals robust narratives of the connection between infertility, sterility, and vaccinations in Zimbabwe and Malawi (Kaler, 2009, 1712). There were also concerns about cholera vaccination in Mozambique. Additionally, in East and West Africa, there was an increase in public concerns regarding vaccinations (Wiysonge, 2019).

In summary, the narratives provided evidence of the influence of religious leadership on the vaccination behavior of individuals and communities. It further shows that it was easier to follow spiritual leaders on health matters because of other factors, including distrust of public health stakeholders like Big Pharma and the Government on issues of race, existential threats via population reduction, and threats to human freedom.

**ii. Poor Theology of Medicines as a Cause of Anti-Vaccine Narratives and Hesitancy:**

In this section, this article asserts that when a theology of medicine is inadequate among Christian leaders, unverified and damaging narratives are inevitable. Such narratives impact adherents' health behavior. The examples and scenarios from Africa and North America will be introduced together, as needed, depending on the subject of discussion. All examples are based on the argument that the poor theology of medicine affects vaccine narratives and related healthcare policies. The following subheadings will present some Neo-Pentecostal theologies that inform healthcare behaviors during the pandemic.

- ***Laying of Hands to cure COVID-19 disease***

In Cameroon, Pastor Frankline Ndifor of Kingship International Ministries downplayed the deadly nature of COVID-19 and claimed he could heal coronavirus sufferers simply by laying hands (The Christian News, 2020; Kindzeka, 2020). Without denigrating the power of the Holy Spirit in healing, and Jesus’s examples in Luke 4:40, imitating Jesus here might not be the best and immediate response.

Two things are critical in laying on hands. One is the expression of great faith, and the other is understanding the nature of diseases brought forward (Dy, 2022). Besides, Jesus mentioned that miracles of this level come with a heavy price, namely prayer and fasting. Jesus did not underestimate the diseases brought forward; there was a comprehensive knowledge of the nature of the disease and quality spiritual preparation. Thus, this article suggests that the theology of laying on of hands may not always be applicable, even during the pandemic. Factors that include understanding the disease, strong spiritual preparation, and developing faith for healing are imperative.

Three reasons may be ascribed to why Pastor Frankline Ndifor died from COVID-19 complications while laying hands on infected patients. First, according to biblical thought, the power, decision, and timing to heal are not his; God retains sovereignty regarding healing (Barry, 2015; cf. Exodus 15:26; Isaiah 53:5; Jeremiah 30:17). Second, there is no evidence that he paid the price as Jesus mentioned to acquire healing powers.

Third, there is no scientific evidence of healing from those prayed for by Pastor Frankline Ndifor at any time. Where there is no scientific evidence in the healing ministry of the pastor, wisdom requires that vaccination be subscribed to as an alternative or in addition to the laying on of hands. This is because healing theology does not exclude the medical profession; God can use medical doctors to heal the sick (Barry, 2015). If a health protocol requires the suspension of laying on of hands to prevent the spread of the virus, then it should be avoided. The same healing hands can spread the virus.

- ***Theology of Death***

Although the latter effort of Pastor Adeboye of the Redeemed Christian Church of God in Nigeria, in encouraging congregants to accept the vaccines, is commendable, his theology of timely death regarding the COVID-19 pandemic may be contestable. In March 2020, Adeboye claimed that **"God is using the coronavirus to prove to the world that he is still almighty God and only those whose time has come will die"** (Obadare, 2022, 1). Additionally, he claims that **"If malaria is killing 200,000 and COVID-19 has not been able to kill 2000, I think malaria is deadlier than COVID-19"** (Obadare, 2022, 1).

Two critical issues are evident in the above claims. First, Adeboye’s reference to the time of death suggests that corona cannot kill anybody except when God’s time to die has come. This submission can aid in hesitating or denying the value of treatment or vaccination. Secondly, the impact of the pandemic was considered lightly compared to malaria. The power of such words from an influential authority in Neo-Pentecostalism can impact congregants' and communities' responses to vaccination. Africans are familiar with suffering from and treating malaria, and if COVID-19, in comparison, is less impactful, why should Africans pay more attention to COVID-19 or consider vaccination a priority?

- ***Theology of Prayer and Fasting***

Charles Blake, the presiding Bishop of the Church of God in Christ (COGIC), in the USA, announced church closures and restrictions after numerous people had died, and others were severely ill and in dire need of interventions. Though the bishop encouraged observance of non-gathering, he did not prioritize vaccination. The bishop claimed, **“Fervent** **prayer** **is our biblical response to any and all societal challenges”** (Quintanilla, 2020,1). Based on this theological position on the pandemic, Blake called for a day of fasting and prayer on March 27, 2020, to mitigate the feared disease.

While prayer is an essential aspect of spiritual care in healthcare, fervent prayer was the only theology emphasized by COGIC leadership as a solution to the COVID-19 pandemic. This theology is challenging, as it may have led congregants to prioritize prayers over vaccination, considering it a secondary concern. Besides COGIC, some American Neo-Pentecostal preachers claimed they can drive out the disease through prayer. Likewise, others continued to engage in supernatural activities to combat the virus, including Kenneth Copeland, the wealthiest pastor in the USA (Elliott, 2020).

- ***The wind of God and Missional Theology as responses to the COVID-19 pandemic***

One of the most dramatic claims of COVID-19 healing was made by Keneth Copeland, who declared that he could blow the virus away with hot air during a broadcast to his followers. Referring to COVID-19, Copeland shouted—**“I blow the wind of God on you! You are destroyed forever, and you will never be back”** (Elliott, 2020, 1). However, after much criticism, the clip no longer appears on his social channels. Another pastor from Louisiana (Tony) brought forward a missional dimension of COVID-19. In his missional theology of the pandemic, Tony states, “**We’re defying the rules because the commandment of God is to spread the Gospel”** (Elliott, 2020, 1). While expecting about 1,000 people to attend his Palm Sunday three services, Tony claims, **“The church is the last force resisting the Antichrist…Let us assemble regardless of what anyone says.”** (Elliott, 2020, 1).

In the claims above, Pastor Tony indirectly considers the COVID-19 pandemic as part of an anti-Christian agenda against the missional assignment of the Church. Further, at least three separate pastors who died from coronavirus think the virus was being used as a tool of the devil to manipulate the masses or silence Christians. One even believes God would use the COVID-19 infection to spread the Gospel or give him a little rest (Blair, 2020). Some American Pentecostals even consider the vaccine as the “mark of the beast” discussed in the last book of the New Testament (McCammon, 2021).

The danger of these theological positions is that they can easily influence congregants, their families, and communities to erect a barrier against considering vaccines or any health protocols provided by health authorities. Compliance with what seems to be anti-missional and antichrist is what Christians will stand against any day. These theological narratives may have contributed to vaccine hesitancy or rejection in North America and Africa.

- ***The Name and the Blood of Jesus as Alternatives to COVID-19 Vaccines***

The Solid Rock Church in Lebanon, Ohio, refused to close after Republican Gov. Mike DeWine urged church leaders to do so. Influenced by the church’s spiritual leader, an attendee became a viral sensation in the media after denouncing the virus threat in a brief interview with CNN (Elliott, 2020). The attendee rejected the reporter’s questions about the dangers of contracting the coronavirus. In the attendee’s words – **“I’m covered in Jesus’ blood”** (Elliott, 2020, 1). The attendee visits grocery stores every day without worrying about contracting an infection. This scenario illustrates the impact of spiritual leaders on the health behaviors of their followers. Regardless of government regulations, members may easily reject vaccines because they have enough vaccines in the name of Jesus.

Additionally, John Hagee of the Cornerstone megachurch claimed that the name of Jesus, the son of the living God, is the vaccine, and doctors responded that such remarks make it more challenging to ensure that all communities are protected from the virus. In a statement to ABC News, Hagee’s ministry clarified that his words were taken out of context because Hagee had taken the vaccine. After all, Hagee believes in the power of prayer and the effectiveness of modern medicine (Vann et al., 2021). The examples provided here demonstrate how the spiritual authority of Neo-Pentecostal leaders, rooted in the theology of the blood and the name of Jesus, significantly influenced COVID-19 vaccine hesitancy and rejection during health crises.

**iii. Role of Social Media and Religious Networks.**

One of North America’s popular Neo-Pentecostal leaders who spent time opposing COVID-19 vaccination was Marcus Lamb, who died of the same virus (BBC, 2021). As an American televangelist, prosperity theologian, Christian broadcaster, and the CEO of Daystar Television Network, Marcus used his media influence to promote the anti-vaccine narrative. The network dedicated hours of broadcasts to anti-lockdown and anti-vaccination activists and groups. His son, Jonathan Lamb, described his father's COVID-19 infection as a spiritual attack from the enemy (Fitzsimons, 2021).

In Canada, Phil Hutchings, the Saint John Church’s founder and lead pastor, who was trained at the Pentecostal Zion Bible College in Rhode Island, posted on Facebook: **“We had a packed service tonight, and it was powerful! But I forgot to tell the public safety authority that we changed locations”** (Smellie, 2021, 1). A humorous emoji followed his Facebook post. This may imply a casual dismissal of COVID-19 protocols and the provincial health policy.

In another scenario, Pastor Landon Spradlin, of Virginia, dismissed the COVID-19 threat as ‘hysteria’ while preaching away from home. Landon fell ill on his way home from the trip and continued to downplay the threat on social media until his death (Elliott, 2020). Likewise, a Canadian megachurch pastor, Leon Fontaine, who refused to adhere to the COVID-19 lockdown and continued holding in-person worship, died at age 59 (Gryboski, 2022). Through a controlling influence over approximately 10,000 regular worshippers in Winnipeg as a televangelist and CEO of Miracle Channel, Leon permits the use of media to promote a narrative about the pandemic, not only to the members but also to the public. Using such media power to promote the anti-vaccine narrative is dangerous to public health and safety**.**

In another case, Bishop Glenn engaged in spiritual bypassing of vaccination in the media. Before dying due to COVID-19 complications, Glenn preached to a few dozen worshipers and posted the sermon on YouTube, which was later removed. As quoted by the local media outlet, Glenn claimed**, “I firmly believe that God is larger than this dreaded virus”** (Vigdor, 2020, 1). The bishop insists on continuous preaching unless in jail or the hospital (Vigdor, 2020). The bishop criticized the government's stay-at-home order during the COVID-19 pandemic. The teaching went viral in local and international media. What the bishop achieved here was a spiritual bypass of vaccination via the spread of the anti-vaccine narrative.

**iv. Poor leadership strategy in Crisis Management**

It cannot be overemphasized that everything rests on leadership. This article proposes that one of the causes of the adverse effects of COVID-19 among Neo-Pentecostals in Africa and America is poor leadership during crises. Available narratives suggest that self-love, as opposed to community love, displayed by spiritual leaders, was at the heart of the increased negative impact of the coronavirus. The case of Neo-Pentecostal leaders taking the initiative to be vaccinated without advising their congregation to do likewise may suggest that leaders would rather protect themselves alone*.*

For example, Leroy Gee, the First United Pentecostal Church pastor in Bishop’s Falls, Newfoundland, Canada, chose not to discuss COVID-19 in sermons because being vaccinated is personal (Barry, 2021). If it is okay to gather for church services, how can the church leadership not encourage everyone in the congregation to get vaccinated? Why did the pastor bypass any conversation about the coronavirus while on the pulpit? Leroy Gee claims to be vaccinated, but telling the congregation to follow suit is unnecessary. Gee says, **“As a minister, I am in town here to help people spiritually”** (Smellie, 2021, 1).

In other words, helping members navigate and survive in health crises is not a priority. Although the clergy may have avoided suggesting to members that they avoid legal implications when vaccine jabs go wrong, such a position may also represent a display of abdicated responsibility rather than a show of leadership in a crisis. Leaders are expected to be anthropological in pastoral leadership.

The biblical principle applicable here is “Love your neighbor as yourself” (Matt. 22:34–40). With this theology, the pastor could have encouraged members to get vaccinated and keep the community safe if the pastor genuinely cared. Gee’s actions may indicate a lack of trust, accountability, and responsibility toward the flock of God and the surrounding community. Gee’s action could have increased the spread and impact of the virus. Like Gee, several Neo-Pentecostal pastors who believe their job over the congregation is only spiritual could have done the same thing during COVID, and thereby increased public health risk.

**v. Influence of Religious Leaders on Adherents’ Health Behavior**.

With their positional authority and microphone, religious leaders wield considerable power and influence over their congregants. Many of the Neo-Pentecostal leaders guided congregants during the pandemic. In Nigeria, the late T.B. Joshua prophesied the end of COVID-19 after the rain in China. Sadly, the prophecy failed. Joshua was not the only clergy member who struggled to get his story about COVID-19 corrected; others who prayed and bound the demonic virus could not succeed. Despite the failed prophecies, most members continued to follow their leaders’ instructions. Thus, the way spiritual leaders influenced the minds of their followers during the pandemic offers insight into the broader role of spirituality in public responses to the epidemic in Nigeria, Africa, and globally (Obadare, 2022).

**Fig. 5:**
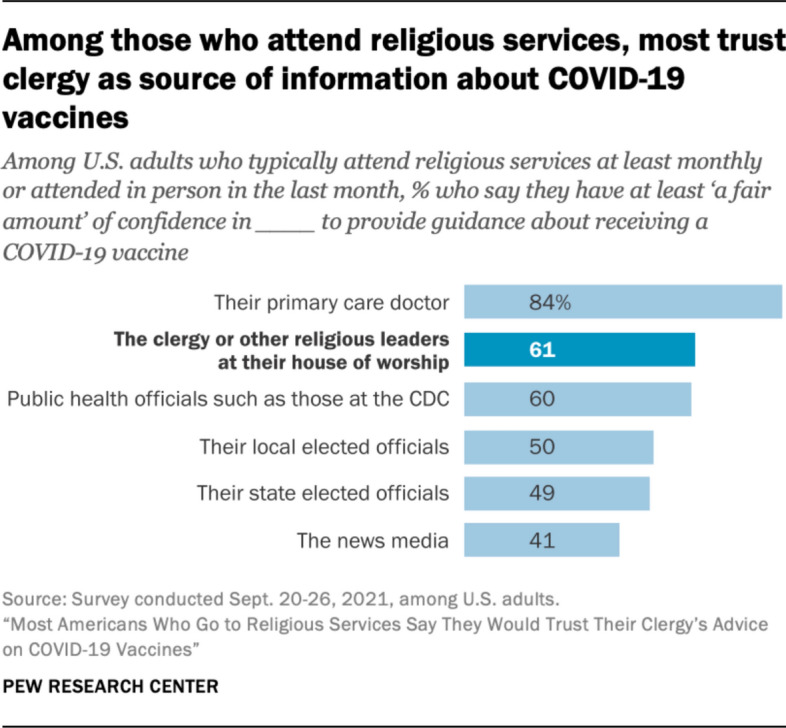
Profession most trusted on COVID-19 decisions. Source: Nortey & Lipka, 2021, Pew Research Centre

Furthermore, Nortey and Lipka, in Pew Research data, assert that approximately 61% of churchgoers in the USA submit to the authority and decisions of their pastors regarding COVID-19 vaccines (Nortey & Lipka, 2021). The implication here is that church adherents highly regard the authority of their spiritual heads. Whether in favor of or opposed to vaccination and health protocols, most faith adherents will follow their leaders’ directives regarding COVID-19. See the statistics in Fig. 5.

The influence of spiritual leaders may have attracted infected patients to them and equally increased the spread of the virus. This has significant public health implications. Pastor Ndifor reportedly died just a week after contracting the disease in Douala, Cameroon. Followers mobbed Ndifor’s house, singing and insisting that the pastor would be resurrected. With many people blocking the entrance to Ndifor’s home, police eventually had to force entry (The Christian News, 2020).

According to the governor of Cameroon’s coastal region, the authorities were called in after the pastor’s death. Still, the followers chased medical staff away, insisting that their leader was on a spiritual retreat with God, not deceased. Medics asked all those who came into close contact with Pastor Ndifor to get tested for COVID-19, as they were at risk of contracting the virus and could also increase its spread. Most of the followers turn down the invitation. Ndifor was so influential that the people never cared about being infected; they clustered around the pastor. Many may have been infected by laying hands, interacting in church, and gathering together after Ndifor’s death. Such actions pose a threat to public health and safety (The Christian News, 2020).

**vi. The healthcare demand and supply gap allows vaccine hesitation and pushback.**

As the world population continues to grow, the demand for healthcare is increasing, while the supply remains limited (Orogun, 2023). Across the globe, demand drives supply not only in economics but also in healthcare. Besides the shortage of healthcare workers, the deterioration of healthcare infrastructure created a vacuum that allowed religious actors to thrive with spiritual diagnoses and cures for biomedical ailments (Obadare, 2022).

Such a demand and supply gap in healthcare has given religious and spiritual healing homes considerable power, causing clients, congregations, and community members to prefer their guidance over that of medical practitioners and government health authorities. This is consistent with the Neo-Pentecostals’ capture of the religious domain and the social imagination of adherents since spiritual leaders represent the first line of protection for many religious people across the globe. Regardless of the non-authenticity and non-scientific narratives, and despite the decisions of the religious leaders, they continue to serve as existential micromanagers who freely pronounce decisions on weightier matters of life and death, such as COVID-19 (Obadare, 2022). In areas where the clergy reject vaccination, hesitancy and pushback are inevitable.

**vii. Consolidation of Pastoral Authority and Economic Gains.**

During the pandemic, the stay-at-home order faced pushback. This may be due to some pastors’ desire to consolidate their authority and disallow activities that could hinder their revenue streams through religious services. The fact that some pastors are forcing people to continually give tithes and offerings during the pandemic may suggest this notion. There have been complaints about how people are losing their minds due to forceful donation demands by some pastors during the pandemic (Ohaja, 2020). As financial interest may be a concern for some pastors, an attempt to stop physical gatherings would make virtual gatherings the alternative. At this level, the fundraising strategies of Neo-Pentecostal leaders may not be as effective as they are in the context of physical gatherings.

Although the government succeeded in closing down the churches in many countries, some Neo-Pentecostal leaders still rebelled against the vaccination as a protest against the lockdown, which works against their economic gains. Others complied with the lockdown order, yet found alternative online means of financial gain. A cyber-ethnographic study of financial gain and management in five selected churches reveals that “the churches' monetary interests and survival strategies were paramount amidst the pandemic” (Nrenzah & Okyerefo, 2022, 1). It can then be inferred that the anti-COVID narratives may be in defense of the economic prosperity of the religious leaders, among other things.

As the lockdown began, pastors cried for help as church cash flow diminished. In Rwanda, Kenya, Nigeria, Ghana, South Africa, and the USA, churches not only complain about the drastic reduction in cash flow but also use the opportunity to demand offerings and healing seeds from congregations (Osei-Kuffour et al., 2022; Rivers & Goldman, 2022; Masson, 2021; Kagire & Buningwire, 2020; Nwafor, 2020; Pararan Mock News, 2020). Meanwhile, some reasonable Neo-Pentecostal leaders rose against those raising money from members and called their actions insensitive, satanic, evil, and greedy (Fosu-Ankrah, 2021).

In consolidating authority, spiritual leaders considered themselves superior to healthcare professionals. They view a concession to a lockdown order as demeaning and inconsistent with maintaining control over congregants (Obadare, 2022). To be consistent with their view of possessing higher authority due to spiritual powers, they had to work against healthcare authorities and preach against the vaccine. In response, some narratives emerged to counter the health authorities.

For example, Bishop Oyedepo and many religious leaders preached that COVID-19 was part of a Western strategy to depopulate Africa, and that the "miraculously" low death rates during COVID-19 in Africa prove that God was on Africa’s side. Oyedepo called on the Nigerian government to visit him to learn how to deal with COVID-19 at no cost (Obadare, 2022). Correspondingly, Reverend Chris Oyakhilome of Christ Embassy, one of the largest Pentecostal churches in the world, told the congregants that the coronavirus is linked to the 5G network and is part of an agenda by a few global leaders.

These spiritual leaders created their narrative to consolidate power and control over their followers’ health behaviors and responses to COVID-19 protocols, thereby generating bias against vaccination. But other Pentecostal leaders, like Poju Oyemade of the Covenant Nation, Sam Adeyemi of the Daystar Christian Centre, both in Lagos, and Matthew Ashimolowo of the Kingsway International Christian Centre, in London, debunked Oyakhilome’s claim (Obadare, 2022).

## Conclusion and Lessons

So far, literature reviews and statistics from academic and media spaces reveal evidence and causes of anti-COVID-19 narratives and hesitancy. The causes influenced the health behavior of Neo-Pentecostal leaders and their followers in Africa and North America. The causes are historical, social, economic, theological, and technological (New Media). Other causes include poor strategic leadership during crises, high demand versus low supply of healthcare, and the consolidation of spiritual authority. The outcome of these narratives is that they offer some valuable lessons, as outlined below.

## Lessons from the Narratives

1. Elements of resistance to pandemic and government control may have emerged prior to the emergence of the COVID-19 narrative. Such a foundation increased current resistance. This has not only affected the acceptance of the COVID-19 vaccine but may also impact future compliance with vaccination in the event of a potential pandemic outbreak.
2. Resistance to vaccination is not only influenced by Christian Religion and Neo-Pentecostal theology and worldview, but it also cuts across religions, as discussed earlier under family planning, polio, and related immunizations.
3. This research underscores that religion is not the only reason behind vaccine hesitancy and resistance; moral, socio-cultural history, and existential issues contribute to the resistance. It illustrates how social, cultural, and racial undertones shape healthcare behavior within a religious context. Although economic factors and the quest to consolidate spiritual authority may be the strongest motivations for the spiritual leaders, public distrust of health stakeholders like Big Pharma and the Government on issues of race, existential threats via population reduction, and threats to human freedom made it easy for the populace to choose their spiritual leaders over healthcare professionals and health authorities. Another significant factor here is the shortage of health care; as demand rises and supply diminishes, it is easier to trust spiritual alternatives.
4. Poor theology of Medicine is behind the dominant and demonizing narratives against vaccines. In addition to poor theology, poor leadership and management skills in pandemic or related crises increase mortality and fatality rates during pandemics.
5. Following poor management skills is the influence of spiritual authority over congregants. Since spiritual authority is a significant factor in determining the health behavior of congregants, it is critical to review when, where, and how to deploy spiritual authority in crises.
6. Literature review reveals that information regarding consultations on the vaccine and related healthcare protocol between the Government health authorities and the Neo-Pentecostal leadership is unpopular in academia, media spaces, and related secondary data. Since the spiritual authority of Neo-Pentecostal leaders influences health behavior, high-level consultation is essential to provide a counter-narrative and promote a positive response to vaccination.

Finally, Part 2 of this topic will cover the public health implications, a robust theological response to the narratives, and additional lessons on how to strategically respond to pandemics from the perspective of religious leadership.

## References

[CR1] Bajeli-Datt, Kavita. (2025). *Is COVID coming back? No need to panic, say docs*. *The New Indian Express*. https://www.newindianexpress.com/nation/2025/May/22/is-covid-coming-back-no-need-to-panic-say-docs

[CR2] Baker, J. O., Perry, S. L., & Whitehead, A. L. (2020). Crusading for moral authority: christian nationalism and opposition to science. *Sociological Forum,**35*(3), 587–607. 10.1111/socf.12619

[CR74] Barry. (2015). *A few thoughts on the theology of Divine healing - Barry Raeburn*. Barry Raeburn. https://www.barryraeburn.org/thoughts-theology-divine-healing/

[CR3] Barry, G. (2021). *Sisters say mother’s death could have been avoided if the church had taken COVID-19 seriously*. CBC. https://www.cbc.ca/news/canada/newfoundland-labrador/covid-19-deaths-bishops-falls-cluster-church-1.6276558

[CR4] Black, M. (2021). *Alberta pastor defiant, refers to “so-called” pandemic on first day of trial for violating public health orders*. CTVNews. https://www.ctvnews.ca/edmonton/article/alberta-pastor-defiant-refers-to-so-called-pandemic-on-first-day-of-trial-for-violating-public-health-orders/

[CR5] Blair, L. (2020). *3 pastors killed by coronavirus; one thought God allowed the infection so he could “get a little rest.”* Christianpost.com; The Christian Post. https://www.christianpost.com/news/3-pastors-killed-by-coronavirus-one-thought-god-allowed-infection-so-he-could-get-a-little-rest.html

[CR7] Carrieri, V., Guthmuller, S., & Wübker, A. (2023). Trust and COVID-19 vaccine hesitancy. *Scientific Reports,**13*(1), Article 9245. 10.1038/s41598-023-35974-z 37286569 10.1038/s41598-023-35974-z PMC10245358

[CR8] Chen, C. (2004). Rebellion against the polio vaccine in Nigeria: Implications for humanitarian policy. *African Health Sciences,**4*(3), 205–207. 15687078 PMC2688336

[CR9] Constantine, N. A., & Jerman, P. (2007). Acceptance of Human Papillomavirus vaccination among Californian parents of daughters: A representative statewide analysis. *Journal of Adolescent Health,**40*(2), 108–115. 10.1016/j.jadohealth.2006.10.007 10.1016/j.jadohealth.2006.10.007 17259050

[CR10] Delehanty, J., Edgell, P., & Stewart, E. (2018). Christian America? secularized evangelical discourse and the boundaries of national belonging. *Social Forces,**97*(3), 1283–1306. 10.1093/sf/soy080

[CR11] Dixon, J. M. (2021). A survey of Covid-19 deaths among American clergy. *Socio-Historical Examination of Religion and Ministry,**3*(2), 238–251. 10.33929/sherm.2021.vol3.no2.03

[CR12] Dy, G. (2022). *Should We Lay Hands on the Sick in Healing?* Christianity.com. https://www.christianity.com/wiki/bible/should-we-lay-hands-on-the-sick-in-healing.html

[CR13] Dyos, S. (2025). *Bill Gates says the Odds of Another Pandemic in the next 4 Years Are 10%–15%, and we’re Not Prepared*. Fortune.com. https://fortune.com/2025/01/26/bill-gates-another-pandemic-odds-covid-not-prepared/

[CR14] Ebrahim, S. H., & Memish, Z. A. (2020). COVID-19: Preparing for superspreader potential among Umrah pilgrims to Saudi Arabia. *Lancet,**395*(10227), Article e48. 10.1016/s0140-6736(20)30466-9 32113506 10.1016/S0140-6736(20)30466-9 PMC7158937

[CR15] Ellingson, C. (2023). *Edmonton-area pastor, church acquitted in Alberta COVID-19 rules case*. CTVNews. https://www.ctvnews.ca/edmonton/article/edmonton-area-pastor-church-acquitted-in-alberta-covid-19-rules-case/

[CR75] Elliott, K. J. (2020). *Rogue pastors deny, “destroy” coronavirus threat ahead of Easter in U.S*. Global News. https://globalnews.ca/news/6787633/coronavirus-easter-pastors/

[CR16] Elliott, Debbie. (2021). *Pastor Who Suffered Through COVID-19 Regrets Not Getting Vaccinated*. NPR. https://www.npr.org/2021/08/24/1030561804/pastor-who-suffered-through-covid-19-regrets-not-getting-vaccinated

[CR17] Fitzsimons, T. (2021). *Marcus Lamb, an anti-vaccine Christian broadcaster, dies after a COVID-19 battle*. NBC News. https://www.nbcnews.com/news/us-news/marcus-lamb-anti-vaccine-christian-broadcaster-dies-covid-battle-rcna7139

[CR18] Fosu-Ankrah, J. F., & Amoako-Gyampah, A. K. (2021). Prophetism in the wake of a pandemic: Charismatic Christianity, conspiracy theories, and the Coronavirus outbreak in Africa. *Research in Globalization,**3*, Article 100068. 10.1016/j.resglo.2021.100068

[CR19] Freimuth, V. S., Jamison, A. M., An, J., Hancock, G. R., & Quinn, S. C. (2017). Determinants of trust in the flu vaccine for African Americans and Whites. *Social Science & Medicine,**193*, 70–79. 10.1016/j.socscimed.2017.10.001 29028558 10.1016/j.socscimed.2017.10.001 PMC5706780

[CR20] Galbraith, K. V., Lechuga, J., Jenerette, C. M., Moore, L. A. D., Palmer, M. H., & Hamilton, J. B. (2016). Parental acceptance and uptake of the HPV vaccine among African-Americans and Latinos in the United States: A literature review. *Social Science & Medicine,**159*, 116–126. 10.1016/j.socscimed.2016.04.028 27180256 10.1016/j.socscimed.2016.04.028 PMC12854015

[CR21] Garenne, M. (2018). Family planning and fertility decline in Africa: from 1950 to 2010. *Family Planning*. 10.5772/intechopen.71029

[CR22] Gorski, P. (2017). Why evangelicals voted for Trump: A critical cultural sociology. *American Journal of Cultural Sociology,**5*(3), 338–354. 10.1057/s41290-017-0043-9

[CR23] Gryboski, M. (2022). *Leon Fontaine, Canadian megachurch pastor, dies at 59*. Christianpost.com; The Christian Post. https://www.christianpost.com/news/leon-fontaine-canadian-megachurch-pastor-dies-at-59.html

[CR24] Howard, K. (2021). *Total Christian Clergy Deaths Resulting from COVID-19*. The FaithX Project. https://faithx.net/2021/07/08/total-christian-clergy-deaths-resulting-from-coronavirus-covid-19/

[CR25] Jaja, I. F., Anyanwu, M. U., & Iwu Jaja, C.-J. (2020). Social distancing: How religion, culture, and burial ceremony undermine the effort to curb COVID-19 in South Africa. *Emerging Microbes & Infections,**9*(1), 1077–1079. 10.1080/22221751.2020.1769501 32459603 10.1080/22221751.2020.1769501 PMC7336991

[CR26] James, A., Eagle, L., Phillips, C., Hedges, D. S., Bodenhamer, C., Brown, R., Wheeler, J. G., & Kirking, H. (2020). High COVID-19 attack rate among attendees at events at a church — Arkansas, March 2020. *MMWR. Morbidity and Mortality Weekly Report,**69*(20), 632–635. 10.15585/mmwr.mm6920e2 32437338 10.15585/mmwr.mm6920e2

[CR27] Jegede, A. S. (2007). What led to the Nigerian boycott of the Polio Vaccination Campaign? *PLoS Medicine,**4*(3), Article e73. 10.1371/journal.pmed.0040073 17388657 10.1371/journal.pmed.0040073 PMC1831725

[CR28] Kagire, E. (2020). *Churches on spotlight for soliciting tithe, offerings amid COVID-19 restrictions*. KT PRESS. https://www.ktpress.rw/2020/03/churches-on-spotlight-for-soliciting-tithe-offerings-amid-covid-19-restrictions/

[CR29] Kaler, A. (2009). Health interventions and the persistence of rumour: The circulation of sterility stories in African public health campaigns. *Social Science & Medicine,**68*(9), 1711–1719. 10.1016/j.socscimed.2009.01.038 19282080 10.1016/j.socscimed.2009.01.038

[CR31] Kindzeka, Moki E. (2020). *Panic grips faithful after cameroon COVID pastor Dies*. Voice of America; Voice of America (VOA News). https://www.voanews.com/a/covid-19-pandemic_panic-grips-faithful-after-cameroon-covid-pastor-dies/6189445.html

[CR76] Liu, J. (2011). *Christian movements and denominations*. Pew Research Center’s Religion & Public Life Project.https://www.pewresearch.org/religion/2011/12/19/global-christianity-movements-and-denominations/

[CR32] Machekanyanga, Z., Ndiaye, S., Gerede, R., Chindedza, K., Chigodo, C., Shibeshi, M. E., Goodson, J., Daniel, F., Zimmerman, L., & Kaiser, R. (2017). Qualitative assessment of vaccination hesitancy among members of the Apostolic Church of Zimbabwe: A case study. *Journal of Religion and Health,**56*(5), 1683–1691. 10.1007/s10943-017-0428-7 28631171 10.1007/s10943-017-0428-7 PMC5711523

[CR77] Majumdar, S. (2022a). *How COVID-19 restrictions affected religious groups around the world in 2020*. Pew Research Center’s Religion & Public Life Project. https://www.pewresearch.org/religion/2022/11/29/how-covid-19-restrictions-affected-religious-groups-around-the-world-in-2020/

[CR33] Majumdar, S. (2022b). *Key findings about COVID-19 restrictions that affected religious groups around the world in 2020*. Pew Research Center. https://www.pewresearch.org/short-reads/2022/11/29/key-findings-about-covid-19-restrictions-that-affected-religious-groups-around-the-world-in-2020/

[CR34] Masson, E. (2021). *Flocks grow, but tithes decline*. The Mail & Guardian. https://mg.co.za/news/2021-04-03-flocks-grow-but-tithes-decline/

[CR35] Mattingly, T. (2020). *GetReligion*. GetReligion. https://www.getreligion.org/getreligion/2020/4/22/washington-post-gets-inside-the-painful-covid-19-crisis-in-church-of-god-in-christ

[CR36] McCammon, S. (2021, April 5). *“Love Your Neighbor” And Get The Shot: White Evangelical Leaders Push COVID Vaccines*. NPR. https://www.npr.org/2021/04/05/984322992/love-your-neighbor-and-get-the-shot-white-evangelical-leaders-push-covid-vaccine

[CR38] National Research Council (US) Working Group on Factors Affecting Contraceptive. (1993). *Factors Affecting Contraceptive Use in Sub-Saharan Africa*. National Academies Press. 10.17226/2209 25144103

[CR39] Nortey, J., & Lipka, M. (2021). *Most Americans who go to religious services say they would trust their clergy’s advice on COVID-19 vaccines*. Pew Research Center’s Religion & Public Life Project. https://www.pewresearch.org/religion/2021/10/15/most-americans-who-go-to-religious-services-say-they-would-trust-their-clergys-advice-on-covid-19-vaccines/

[CR40] Nrenzah, G., & Okyerefo, M. P. K. (2022). Religion and commodification: The Ghanaian Churches’ COVID-19 economy. *Ghana Journal of Religion and Theology,**11*(1–2), 133–155. 10.4314/gjrt.v11i1-2.9

[CR41] Nwafor. (2020). *South-South pastors disagree over “Covid-19 money.”* Vanguard News. https://www.vanguardngr.com/2020/05/south-south-pastors-disagree-over-covid-19-money/

[CR42] O’Donnell, K. (2020). The case against the doctors: Gender, authority, and critical science writing in the 1960s. *Journal of the History of Medicine and Allied Sciences,**75*(4), 429–447. 10.1093/jhmas/jraa028 32869099 10.1093/jhmas/jraa028

[CR43] Obadare, E. (2022). *The Spiritual Dimension of COVID-19 in Africa | Think Global Health*. Think Global Health. https://www.thinkglobalhealth.org/article/spiritual-dimension-covid-19-africa

[CR44] Ohaja, E. (2020). *On complaints against collection of tithes and offerings during COVID-19 Pandemic – Edith Ohaja*. Edithohaja.com. https://edithohaja.com/on-complaints-against-tithes-offerings-during-covid-19-pandemic/

[CR45] Orogun, D. (2023). *Improving spiritual care to bridge the gap between demand and supply of healthcare services in South Africa*. Scientia Moralitas Research Institute. https://www.ceeol.com/search/chapter-detail?id=1210451

[CR46] Orubuloye, I. O., Caldwell, J. C., & Caldwell, P. (1993). The role of religious leaders in changing sexual behavior in Southwest Nigeria in an era of AIDS. *Health Transition Review,**3*, 93–104. 10.2307/40652064

[CR47] Osei-Kuffour, F., Peprah, W. K., Sarfo, D. M., & Yeboah, B. O. (2022). COVID-19 impact on church cash inflows in Ghana as moderated by location profile. *Applied Finance and Accounting,**8*(1), 6–6. 10.11114/afa.v8i1.5736

[CR48] Pararan Mock News. (2020). *Pastor Oyedepo is still asking for tithes during corona_virus lockdown (Mock News )(Comedy News)*. YouTube. https://www.youtube.com/watch?v=q70GObsvQzE

[CR49] Peagler, A. (2020). *COVID-19 has killed several bishops and pastors within the Church of God and Christ, headquartered in Memphis*. Localmemphis.com; WATN. https://www.localmemphis.com/article/news/health/coronavirus/covid-19-has-killed-several-bishops-and-pastors-within-church-of-god-and-christ-headquartered-in-memphis/522-bf2930fe-0e10-42a4-b105-d0d8dfe5e80d

[CR51] Poitras, J. (2021). *“Jesus was a healer”: One man’s rage at anti-vaccination pastors in local churches*. CBC. https://www.cbc.ca/news/canada/new-brunswick/covid-19-church-perth-andover-1.6194479

[CR53] Quintanilla, M. (2020). *Up to 30 Bishops, Pastors Have Died from COVID-19 in the Largest Black Pentecostal Denomination*. Crosswalk.com; Crosswalk. https://www.crosswalk.com/headlines/contributors/milton-quintanilla/up-to-30-bishops-pastors-have-died-from-covid-19-in-largest-black-pentecostal-denomination.html

[CR54] Renne, E. (2006). Perspectives on polio and immunization in Northern Nigeria. *Social Science & Medicine,**63*(7), 1857–1869. 10.1016/j.socscimed.2006.04.025 16765498 10.1016/j.socscimed.2006.04.025

[CR55] Rivers, A., & Goldman, S. (2022). *Black Churches and the Pandemic • Disaster Recovery Journal*. Disaster Recovery Journal. https://drj.com/journal_main/black-churches-and-the-pandemic/

[CR56] Sheel, Meru . (2025, May 7). *COVID is still around and a risk to vulnerable people. What are the symptoms in 2025? And how long does it last?* The Conversation. https://theconversation.com/covid-is-still-around-and-a-risk-to-vulnerable-people-what-are-the-symptoms-in-2025-and-how-long-does-it-last-253840

[CR57] Sisti, L. G., Buonsenso, D., Moscato, U., Costanzo, G., & Malorni, W. (2023). The role of religions in the COVID-19 pandemic: A narrative review. *International Journal of Environmental Research and Public Health,**20*(3), Article 1691. 10.3390/ijerph20031691 36767057 10.3390/ijerph20031691 PMC9914292

[CR58] Smellie, S. (2021). *Pentecostal churches in Atlantic Canada under scrutiny as push to vaccinate ramps up*. CityNews Halifax. https://halifax.citynews.ca/2021/10/14/pentecostal-churches-in-atlantic-canada-under-scrutiny-as-push-to-vaccinate-ramps-up-4514564/

[CR59] Sokolow, A. (2020). *With science and scripture, a Baltimore pastor is fighting Covid-19 vaccine skepticism*. STAT. https://www.statnews.com/2020/08/31/with-science-and-scripture-a-baltimore-pastor-is-fighting-covid-19-vaccine-skepticism/

[CR60] The Christian News. (2020). *African pastor who claimed to heal people of COVID-19 dies from the virus*. Premierchristian.news; Premier Christian News. https://premierchristian.news/en/news/article/african-pastor-who-claimed-to-heal-people-of-covid-19-dies-after-contracting-the-virus

[CR61] Unfried, K., & Priebe, J. (2024). Vaccine hesitancy and trust in sub-Saharan Africa. *Scientific Reports,**14*(1), Article 10860. 10.1038/s41598-024-61205-0 38740790 10.1038/s41598-024-61205-0 PMC11091197

[CR62] Vann, M., Kaitlyn, F., Hosenball, A., & Jung, J. (2021). *Blessing by way of medicine: These pastors preach COVID-19 vaccination as God’s healing power*. ABC News. https://abcnews.go.com/Politics/blessing-medicine-pastors-preach-covid-19-vaccination-gods/story?id=76367456

[CR63] Vigdor, N. (2020). Pastor who defied social distancing dies after contracting Covid-19, Church Says. *The New York Times*. https://www.nytimes.com/2020/04/14/us/bishop-gerald-glenn-coronavirus.htm

[CR64] Washington, H. A. (2008). *Medical Apartheid by Harriet A. Washington: 9780767915472 | PenguinRandomHouse.com: Books*. PenguinRandomhouse.com. https://www.penguinrandomhouse.com/books/185986/medical-apartheid-by-harriet-a-washington

[CR65] Webb, N. S., Dowd-Arrow, B., Taylor, M. G., & Burdette, A. M. (2018). Racial/ethnic disparities in influenza vaccination coverage among US adolescents, 2010-2016. *Public Health Reports,**133*(6), 667–676. 10.1177/003335491880572 30300560 10.1177/0033354918805720 PMC6225871

[CR66] Whitehead, A. L., & Perry, S. L. (2020). How culture wars delay herd immunity: Christian nationalism and anti-vaccine attitudes. *Socius: Sociological Research for a Dynamic World,**6*, Article 237802312097772. 10.1177/237802312097772

[CR67] Wise, J. (2001). Pfizer accused of testing new drug without ethical approval. *BMJ,**322*(7280), 194–194. 10.1136/bmj.322.7280.19 11159610 PMC1119465

[CR68] Wiysonge, C. S. (2019). *Vaccine Hesitancy, an Escalating Danger in Africa | Think Global Health*. Council on Foreign Relations. https://www.thinkglobalhealth.org/article/vaccine-hesitancy-escalating-danger-afric

[CR71] Yillah, R. M., Bull, F., Sawaneh, A., Reindorf, B., Turay, H., Wurie, H. R., Hodges, M. H., & Osborne, A. (2024). Religious leaders’ nuanced views on birth spacing and contraceptives in Sierra Leone – qualitative insights. *Contraception and Reproductive Medicine*, *9*(1). 10.1186/s40834-024-00301- 10.1186/s40834-024-00301-y PMC11342541 39180098

[CR72] YouVersion. (2025). *3 John 1:2*. YouVersion | the Bible App | Bible.com; YouVersion. https://www.bible.com/bible/3034/3JN.1.2.BSB

[CR73] Zewoldi, Y., & Eca, U. (2025). *Fertility behavior of elites and their perception of the population problem in Ethiopia: a synthesis /: by Yacob Zewoldi.* United Nations Digital Library System; UN, ECA. https://digitallibrary.un.org/record/187010?ln=en

